# Trends in antidepressant prescriptions in children and young people in England, 1998–2017: protocol of a cohort study using linked primary care and secondary care datasets

**DOI:** 10.1136/ebmental-2019-300097

**Published:** 2019-06-28

**Authors:** Ruth H Jack, Chris Hollis, Carol Coupland, Richard Morriss, Roger David Knaggs, Andrea Cipriani, Samuele Cortese, Julia Hippisley-Cox

**Affiliations:** 1 Division of Primary Care, University of Nottingham, Nottingham, UK; 2 Division of Psychiatry and Applied Psychology, University of Nottingham, Nottingham, UK; 3 National Institute for Health Research (NIHR) Nottingham Biomedical Research Centre, Nottingham, UK; 4 National Institute for Health Research (NIHR) Mental Health MedTech Co-operative (MindTech), Nottingham, UK; 5 School of Pharmacy, University of Nottingham, Nottingham, UK; 6 Department of Psychiatry, University of Oxford, Oxford, UK; 7 Centre for Innovation in Mental Health, University of Southampton, Southampton, UK; 8 Clinical and Experimental Sciences (CNS and Psychiatry), University of Southampton, Southampton, UK; 9 Child Study Center, New York University, New York, NY, USA; 10 Nuffield Department of Primary Care Health Sciences, University of Oxford, Oxford, UK

## Abstract

**Introduction:**

Increasing numbers of children and young people (CYP) are receiving prescriptions for antidepressants. This is the protocol of a study aiming to describe the trends and variation in antidepressant prescriptions in CYP in England, and to examine the indications for the prescriptions recorded and whether there was contact with secondary care specialists on or around the time of the first antidepressant prescription.

**Methods and analysis:**

All eligible CYP aged between 5 and 17 years in 1998–2017 from the QResearch primary care database will be included. Incidence and prevalence rates of any antidepressant prescription in each year will be calculated. We will examine four different antidepressant classes: selective serotonin reuptake inhibitors, tricyclic and related antidepressants, serotonin and norepinephrine reuptake inhibitors and other antidepressants, as well as for individual drugs. Linked primary and secondary care data (hospital episode statistics) in the year before and up to 6 months after the first antidepressant prescription will be examined for CYP whose first antidepressant prescription was in 2006–2017. Whether there were records of indications and being seen by psychiatric or paediatric specialists will be identified. Trends over time and differences by region, deprivation and ethnicity will be examined using Poisson regression.

**Discussion:**

This large, population-based study will give an up-to-date picture of antidepressant prescribing in CYP and identify any variation. Understanding what indications are recorded when CYP are being prescribed antidepressants, and whether this was done in partnership with secondary care specialists, will provide evidence of whether appropriate guidelines are being followed.

## Introduction

In a UK 2017 national survey,^1^ the estimated proportions of children and young people (CYP) in England with depressive disorders were 0.3% of 5–10 year-olds, 2.7% of 11–16 year-olds and 4.8% of 17–19 year-olds. These figures reflect around 200 000 affected 5–19 year-olds.^2^ The proportion of 5–15 year-olds with depressive disorders in England was estimated to have increased from 0.9% in 1999 and 0.8% in 2004 to 1.2% in 2017. Recording of depression symptoms rather than diagnoses is higher, and reported by 24% of girls and 9% of boys aged 14 in the UK-based Millennium Cohort Study.^3^ Compared with adults, children and adolescents with major depressive disorder are still underdiagnosed and undertreated,^4^ possibly because they tend to present with rather undifferentiated depressive symptoms, for example, irritability, aggressive behaviours or school refusal.^5^ Consequences of depressive episodes in these young people include serious impairments in social functioning, as well as suicidal ideation and attempts.^6^

Although psychological treatments are considered the first-line treatment in many clinical guidelines including the UK The National Institute for Health and Care Excellence (NICE) guidelines,^6^ antidepressants are widely used in the treatment of depression in children and adolescents, and in the past few decades prescription of antidepressants in young people has increased significantly both in the UK^7–9^ and in other countries.^10^ However, even though antidepressants in combination with psychological therapy is recommended for moderate to severe depression in the NICE guideline (CG28) for depression in CYP,^11^ the efficacy and safety of antidepressant medicines remain controversial.^12 13^ In line with these recommendations and based on results of meta-analyses,^13^ the only first-line antidepressant recommended for CYP with moderate to severe depression is fluoxetine initiated in secondary care following assessment and diagnosis by a child and adolescent psychiatrist.^6^ Sertraline and citalopram are recommended by NICE as second-line antidepressants in particular circumstances if fluoxetine is not tolerated or unsuccessful.^11^ Some antidepressants can be prescribed to CYP with obsessive-compulsive disorder (alongside psychological treatments)^14^ or for other conditions,^15^ including unlicensed ‘off-label’ use, such as for neuropathic pain.^16^

Studies have shown a pattern of increasing antidepressant prescription rates in CYP in the UK, up to 2009,^7^ 2011,^8^ 2013^17^ and 2015.^9^ Rates of depression symptom recording have increased while recorded diagnoses of depression have decreased over the same periods.^17^ This pattern of symptoms rather than diagnoses being recorded is demonstrated by previous work in the UK finding only 24% of those first prescribed antidepressants had a depression diagnosis within the previous year to 4 weeks after the first prescription,^9^ while another study showed over half had either a depression diagnosis or depression symptoms recorded in the year before to 6 months after their prescription.^17^

So far, there has been little research into variation in antidepressant prescribing, particularly in CYP. Recent evidence shows that rates of antidepressant prescriptions in England in 2015/2016 were lowest in London, and highest in the North East.^18^ Studies have shown that children and adolescents^7 17^ and adults^18^ residing in the most deprived quintiles were more likely to receive antidepressant prescriptions compared with those in the least deprived quintile in the UK.

Surveys found that Black, South Asian and Mixed/other ethnic groups were less likely to have received antidepressants than the White group.^19^ Within areas of London, antidepressant use was highest in the White British group (8.1%), almost half this rate in Bangladeshis (4.4%) and the lowest rate was in the Indian and Black African groups (2.1%).^20^ While these studies used self-assigned person-level ethnicity, others have used different measures of ethnicity. A study using the proportion of Asian names on general practitioner (GP)’s practice lists found antidepressant prescribing was higher in practices with low proportions of Asians on the list,^21^ and another showed antidepressant prescription volumes were lower in GP practices with high densities of Black and South Asian populations.^22^

The present study provides an opportunity to examine variation in antidepressant prescriptions in CYP by deprivation, region and ethnicity simultaneously in a large population from England, over a 20-year period. This will allow any inequalities to be identified, and enable future studies to determine whether these are due to differences in need or due to inequity, where any differences are avoidable and unfair.

Our study aims to (1) examine changes over time and variation in antidepressant prescribing in CYP aged between 5 and 17 years old between 1998 and 2017, (2) investigate the clinical indications associated with these prescriptions recorded in primary and secondary care data and (3) assess adherence with NICE guideline recommendations.

Our objectives are as follows:

- To describe differences in antidepressant prescribing over time by age, sex, region, deprivation and ethnicity.
- To compare patterns of antidepressant prescribing against NICE guideline recommendations for the management of depression in CYP to determine whether fluoxetine is used as a first-line treatment.
- To determine whether those with antidepressant prescriptions have the appropriate clinical indications recorded in primary or secondary care records.
- To examine admitted patient care and outpatient hospital episode statistics (HES) data to identify whether CYP had been assessed in secondary care by a child and adolescent psychiatrist (as recommended by NICE CG28) at the time of initiation of antidepressant prescribing.

## Methods and analysis

### Patient and public involvement

This work is part of the National Institute for Health Research Nottingham BRC Mental Health & Technology theme looking at decision aid tools and risk assessment. Patient and public involvement (PPI) representatives are part of this research team, attending monthly project meetings and additional, more frequent, meetings focusing on PPI aspects of the project.

### Study design and data sources

We will examine incidence and prevalence of antidepressant prescribing in a cohort of CYP in England using a large primary care database (QResearch, version 43) linked to HES admitted patient care and outpatient data. At the time of the study, the QResearch database includes health records of over 32 million people from more than 1500 general practices across the UK which record data using the Egton Medical Information Systems (EMIS) medical records computer system. The information recorded includes personal characteristics, clinical diagnoses, symptoms and prescribed medicines.

The study cohort will include people who were aged between 5 and 17 years between 1 January 1998 and 31 December 2017. Each person’s study entry date is defined as the latest date of 12 months after their registration with a study practice, 12 months after the installation date of their practice’s EMIS computer system, 1 January of the year they turned 5 years old or 1 January 1998. People will then be followed up until the earliest date of them leaving the practice, dying, 1 January of the year they turned 18 years old or the end of the follow-up period (31 May 2018).

We will examine trends separately for males and females, as defined in their primary care data. Overall trends will be examined for those aged 5–11 and 12–17 years—similar to the age groups specified in the NICE guidelines on depression in children and young people,^ 11^ but excluding 18-year-olds who may have been treated as adults. We will also study trends over time for different regions of England, Townsend deprivation quintiles and ethnic groups. Where ethnicity information is missing in QResearch, we will supplement this with the most recent valid ethnic code available in HES. We will examine the five broad ethnic groups as used in the England and Wales 2001 Census: White, Mixed, Asian or Asian British, Black or Black British, and Chinese or other ethnic group, plus those with no known ethnicity.

We will examine information on prescriptions for any antidepressant within the study period, and analyse the overall incidence and prevalence of antidepressant prescribing by year. Those with a record of an antidepressant prescription before their study entry date will be excluded from the incidence cohort. We will also examine four different drug classes: selective serotonin reuptake inhibitors (SSRIs), tricyclic and related antidepressants (TCAs), serotonin and norepinephrine reuptake inhibitors (SNRIs) and other antidepressants. In addition, we will examine individual antidepressant drugs separately. Antidepressants included in the different drug classes are shown in table 1.

**Table 1:** Antidepressants to be included in each drug class

SSRIs	TCAs	SNRIs	Other
Citalopram	Amitriptyline	Duloxetine	Agomelatine
Escitalopram	Amoxapine	Venlafaxine	Flupentixol*
Fluoxetine	Butriptyline	Pristiq†	Isocarboxazid
Fluvoxamine	Clomipramine		L-Tryptophan
Paroxetine	Desipramine		Mirtazapine
Sertraline	Dosulepin		Moclobemide
	Doxepin		Nefazodone
	Imipramine		Phenelzine
	Iprindole		Reboxetine
	Lofepramine		Tranylcypromine
	Maprotiline		Tryptophan
	Mianserin		Vortioxetine
	Nortriptyline		Wellbutrin†
	Protriptyline		
	Trazodone		
	Trimipramine		
	Viloxazine		

*An antipsychotic prescribed at lower doses for depressive illness in adults and the elderly. Therefore, only 1 mg and 500 μg tablets will be included.

†Brand name given as generic name is not included in search.

SNRIs, serotonin and norepinephrine reuptake inhibitors; SSRIs, selective serotonin reuptake inhibitors; TCAs,  tricyclic and related antidepressants.

Indications recorded and whether CYP were seen by a secondary care specialist around the time of the first antidepressant prescription will be investigated. This analysis will be limited to CYP who had their first antidepressant prescription in 2006–2017. This period is after the NICE guideline for depression in CYP was published in 2005,^11^ and the quality and outcomes framework, which rewards general practices in the UK, introduced an indicator relating to assessing severity in patients with a new depression diagnosis in 2006.^23^ Diagnostic Read codes, which are the standard clinical codes used in general practice in the UK, will be used to identify CYP with a diagnosis of depression, any symptoms around depression, anxiety, phobias, obsessive-compulsive disorder, neuropathic pain, enuresis and self-harm and also whether they had been referred to mental health services. We will use codes to identify depression diagnoses that have been employed in previous studies.^24 25^ We will also use F32 (depressive episode), F33 (recurrent depressive disorder), F40 (phobic anxiety disorders), F41 (other anxiety disorders), F42 (obsessive-compulsive disorder), F93.0 (separation anxiety disorder of childhood), F93.1 (phobic anxiety disorder of childhood), F93.2 (social anxiety disorder of childhood) and X60-X84 (intentional self-harm) codes from the International Classification of Diseases V.10 (ICD-10) to identify indications using the linked HES datasets. We will examine whether there are records of these indications and of referral to mental health services either in the year before or up to 6 months after their first antidepressant prescription, apart from self-harm indications where we will only include those recorded up to a year before the first antidepressant prescription. We will examine the specialty of the consultant from admitted patient and outpatient HES data to identify if the CYP had contact with child and adolescent psychiatry, forensic psychiatry, psychotherapy, adult mental illness, paediatrics or paediatric neurology consultants either in the year before or up to 6 months after their first antidepressant prescription.

### Statistical analysis

We will calculate incidence rates per 1000 person-years for being first prescribed an antidepressant to examine trends over time between 1998 and 2017 in males and females aged between 5 and 17 years. We will also calculate prevalence rates per 1000 person-years over time for people with any prescriptions for antidepressants, whether these were first or subsequent prescriptions. These will be produced overall and for the separate subgroups described above. Of those who had been prescribed antidepressants, we will calculate the proportions first prescribed different antidepressant groups and individual drugs. Incidence rate ratios will be calculated using Poisson regression for the different subgroups in the different age and sex groups over time, clustered by GP study practice. In order to take account of varying patterns in antidepressant prescribing over time, linear trends will be assessed for different periods using piecewise linear regression^26^ with change points identified by previous studies: 2002 and 2005.^8 12^ Mars *et al* 8 also identified 2008 as a change point for antidepressant prevalence in people aged ≥14, but not incidence. We will include this too and assess its significance, as our study has a longer follow-up than the original analysis. These time points relate to different factors that were likely to have influenced antidepressant prescribing, particularly in CYP. In 2003, concerns around increased suicidal behaviour in association with paroxetine were raised, leading the UK medicines and healthcare products regulatory agency to contraindicate first paroxetine,^27^ then venlaflaxine^28^ and finally all SSRIs except fluoxetine^29^ in under 18s. Antidepressant prescriptions would therefore have peaked in 2002. In the UK, NICE Guideline CG28 on the management of depression in CYP was first published in 2005,^11^ and an update of the British Association for Psychopharmacology guidelines for treating depressive disorders with antidepressants was published in 2008.^30^

For people who were newly prescribed antidepressants in 2006–2017, we will examine what proportion had received a diagnosis of depression, anxiety, obsessive-compulsive disorder, neuropathic pain or enuresis, or had symptoms of depression or self-harm indications recorded. We will determine what proportion of CYP had been seen by appropriate secondary care consultants between a year before and 6 months after their first prescription for antidepressants. We will produce these analyses for any antidepressant, the main antidepressant groups and the most commonly prescribed individual drugs. This will be done separately by age, sex, region, Townsend deprivation quintiles, and ethnic group, where numbers are sufficient.

## Discussion

In this study, we will examine the incidence and prevalence of antidepressant prescribing in CYP in England over a 20-year period. In order to identify inequalities, we will study variation in prescribing rates by region, deprivation and ethnicity. We will examine the indications recorded in primary care data for the first antidepressant prescription, and whether there is evidence of appointments with appropriate secondary care consultants. The QResearch and HES data needed for the project have already been collected and linked. Many of the Read code groups for indications have been used in previous studies, while others will be created and approved by the study team.

### Strengths and limitations

There are several strengths of our study. Examining data from a period of 20 years will allow long-term patterns to be assessed, in the context of published guidance and warnings that are likely to have influenced antidepressant prescribing. A large sample size will allow variation to be examined by region, deprivation and ethnic groups, adjusted for other factors. The use of both primary and secondary care data will give a fuller picture of the indications and specialists involved in antidepressant prescribing in CYP. A limitation will be that we will only be able to examine primary care prescriptions. Any prescribing solely within secondary care will not be included as it is not routinely recorded in GP records, and it’s possible that we will therefore miss the true first antidepressant prescription. Only year of birth is recorded in QResearch to preserve anonymity. By taking 1 January as date of birth, we will include some 4-year-olds and exclude some 17-year-olds from the cohort. This means our estimates of antidepressant prescriptions will be slight underestimates, as usage increases with age. It is possible that we will overestimate antidepressant use, as CYP may not take the antidepressants they were prescribed. However, we will be able to examine prescribing patterns, and by only assessing whether or not antidepressants were prescribed rather than how many prescriptions in the prevalence analysis, this is likely to be minimised. It is expected that some records will have inaccurate or incomplete information recorded. By determining what proportion of patients do not have appropriate information recorded, we will be able to evaluate the scale of these problems. This will highlight the need for improved recording, to ensure continuity of care between healthcare professionals.

## Conclusion

This study will give an up-to-date picture of antidepressant prescribing in CYP and whether this varies by region, deprivation or ethnic group. Identifying any variation will allow future work to focus on whether this is due to differences in need or practice. We will also examine primary and secondary care data to determine what indications are recorded associated with initial antidepressant prescriptions, and whether CYP were seen by appropriate secondary care specialists. Understanding whether CYP are being prescribed antidepressants for licensed or unlicensed indications, and whether this was done in partnership with secondary care specialists, will provide evidence of whether appropriate guidelines are being followed.

### Ethics and dissemination

The project has been reviewed in accordance with the QResearch agreement with East Midlands—Derby Research Ethics Committee [reference 18/EM/0400]. To guarantee the confidentiality of patient information, only the authors will have access to the data during the study. Two articles are planned to be submitted for publication from this work: (1) trends and variation in the incidence and prevalence of antidepressant prescriptions over time and (2) recording of indications and secondary care specialists seen around the time of first antidepressant prescription. The full statistical code will be available from the authors after the publication of the results.
